# Prognostic Significance of Subtype Classification for Short- and Long-Term Survival in Breast Cancer: Survival Time Holds the Key

**DOI:** 10.1371/journal.pmed.1000281

**Published:** 2010-05-25

**Authors:** Stefan Ambs

**Affiliations:** Laboratory of Human Carcinogenesis (LHC), Center for Cancer Research (CCR), National Cancer Institute (NCI), Bethesda, Maryland, United States of America

## Abstract

Stefan Ambs provides a perspective on a recent research article by Paul Pharoah and colleagues that evaluated the prognostic significance of immunohistochemical subtype classification in early breast cancer.

**Linked Research Article**

This Perspective discusses the following new study published in *PLoS Medicine*:

Blows FM, Driver KE, Schmidt MK, Broeks A, van Leeuwen FE, et al. (2010) Subtyping of Breast Cancer by Immunohistochemistry to Investigate a Relationship between Subtype and Short- and Long-Term Survival: A Collaborative Analysis of Data for 10,159 Cases from 12 Studies. PLoS Med 7(5): e1000279. doi:10.1371/journal.pmed.1000279

Paul Pharoah and colleagues evaluate the prognostic significance of immunohistochemical subtype classification in more than 10,000 breast cancer cases with early disease, and examine the influence of a patient's survival time on the prediction of future survival.

## “We still lack the ability to accurately predict survival among breast cancer patients with early-stage disease”

Advances in hormone therapy and combination chemotherapy have led to significant declines in disease recurrence and mortality due to breast cancer [1],[2]. However, the burden of developing a deadly disease remains unequal. A disease diagnosis at age 50 or younger is still more likely to have a fatal outcome than the same diagnosis at an older age. Moreover, African-American women and women diagnosed with estrogen, progesterone, and HER2 receptor–negative disease (termed triple-negative breast cancer) continue to experience an excessive mortality and have not benefited from the advances in breast cancer therapy as much as other patient groups [3],[4]. Currently, about 10% of breast cancer patients will succumb to the disease within the first 5 years following the initial diagnosis [5], despite many being diagnosed with an early-stage disease. The relative risk of dying from breast cancer has a peak between 2 and 4 years post-diagnosis [2], and we can only speculate why so many women die in that period. It remains a great challenge to prospectively identify those breast cancer patients who have been diagnosed with a primary disease but have a low chance of surviving when treated with current standard therapy.

A study by Paul Pharoah and colleagues [6] published in this week's *PLoS Medicine* evaluated immunohistochemistry-based subtype classification of breast tumors for prediction of disease outcome. The authors recommend that this method is being used in clinical practice. Yet, questions remain of how useful subtype classification will be in the management of breast cancer.

## Breast Cancer Molecular Subtypes Are Biologically Distinct and Have Different Outcomes

Landmark studies by Perou et al. and by Sorlie et al. identified five distinct subtypes, which are different from one another in their gene expression characteristics [7],[8]. They include two estrogen receptor (ER)-positive subtypes, termed luminal A and B, and three ER-negative subtypes, termed basal-like, HER2-positive, and normal breast-like tumors. Luminal tumors develop more frequently than the other subtypes and share many features with luminal epithelial cells lining the mammary ducts, but tumors in the luminal B subgroup also aberrantly overexpress the HER2 receptor or proliferation marker genes that distinguish them from the HER2-negative luminal A tumors. Basal-like breast cancer has similarities with normal breast basal epithelial cells and is commonly identified by a lack of ER and HER2 receptor expression and by the expression of either cytokeratin 5/6 or epidermal growth factor receptor (EGFR). This group of tumors is characterized by a high proliferation rate, pushing border of invasion, and the frequent expression of cancer stem cell markers [9]. Basal-like breast tumors share many features with triple-negative tumors although the overlap is not complete, raising the question of whether they should be treated as two different entities in clinical practice. A diagnosis of either the basal-like or triple negative disease signals a diminished chance of survival because of high relapse rates within the first three years after diagnosis, mainly affecting patients with residual disease despite the use of adjuvant chemotherapy.

## Prognostic Significance of Subtype Classification and the Effect of Survival Time

Numerous studies have shown that immunohistochemistry for hormone and EGFR receptors and cytokeratin 5/6 expression is a simple and workable way to determine the intrinsic subtype of breast tumors for clinical applications [10],[11]. Moreover, immunohistochemical classification into either a luminal A, HER2-expressing, basal-like, or triple-negative tumors was found to be associated with a patient's survival, and tumor subtypes were shown to respond differently to adjuvant therapy, with many basal-like and HER2-positive tumors being particularly sensitive to anthracycline-based combination therapy [12],[13]. These findings suggested that immunohistochemistry-based classification of breast tumors may improve prognostication and a patient's assignment to therapy beyond current standards in breast cancer care. However, several studies challenged this paradigm and signs emerged that categorization of breast tumors into good and poor outcome groups based on their intrinsic subtype classification may have limitations. Currently unknown factors seem to further separate several of the intrinsic subtypes into two clinically distinct subgroups: one with patients who succumb to the disease early and one with patients who continue to survive the disease [11].

The analysis by Pharoah and colleagues published in *PLoS Medicine* evaluated the prognostic significance of immunohistochemical subtype classification in over 10,000 cases with early disease, examining the influence of a patient's survival time on the prediction of future survival [6]. The authors subtyped breast tumors into HER2-negative and HER2-positive luminal tumors, HER2-positive ER-negative tumors, tumors with the core basal phenotype (“basal-like”) and tumors that lacked expression of the estrogen, progesterone, and HER2 receptors and also cytokeratin 5/6 and EGFR (“triple-negative tumors that are not basal-like”). Consistent with existing literature, short- and long-term survival of breast cancer patients was found to differ by subtype, arguing that subtype classification is clinically useful and will help determine appropriate therapies. The authors also provided more evidence that basal-like tumors are clinically distinct from triple-negative tumors.

However, in contrast to many previous reports, a simple categorization of basal-like, triple-negative, or HER2-positive tumors as poor-outcome subtypes is not supported by this new study. Instead, initial differences in survival between subtypes were found to change with increasing survival time, and all previously described poor-outcome subtypes, when compared to ER-positive, HER2-negative tumors, became in fact good outcome subtypes if patients survived longer than five years. The observed survival patterns were independent of any systemic adjuvant therapy, suggesting that tumor biology and molecular heterogeneity within breast cancer subtypes, rather than the choice of therapy, determined the survival trends.

How robust are the findings from this new study? The strength of the study is clearly its size and the careful analysis of differences in patient survival by tumor subtype including the calculation of period-specific hazard ratios. However, some limitations may arise from the use of pooled data from 12 independent studies, possibly introducing heterogeneity and misclassification errors, the predominance of ER-positive, HER2-negative tumors in the study population, and the limited racial/ethnic diversity of the patients. Nevertheless, these limitations should not challenge the validity and broad implication of the findings.

## Is There a Limitation to Subtype Classification for Prognostication and Targeted Therapy?

Pharoah and colleagues describe a pattern for short- and long-term breast cancer survival that reflects a constant mortality rate for ER-positive, HER2-negative tumors and a bimodal mortality rate pattern for all other breast cancer subtypes, with an initially high mortality rate that progressively declines. Changes in mortality rates and disease prognosis with increasing survival time will complicate decision making in clinical practice. What causes mortality rates to change when patients survived the first years after diagnosis? It is partly explained by the excess risk of early relapse, leading to excess mortality within the first five years, for patients that have residual disease and have been diagnosed with a basal-like, triple-negative, or HER2-positive tumor. It appears that these relapsing patients cannot be saved by current standard therapy. It is a limitation of the immunohistochemistry approach applied by Pharoah and colleagues, and of the intrinsic breast cancer subtype model in general, that it cannot differentiate within a subtype between those patients who will succumb to the disease within a few years and those who will continue to survive the disease. It is unlikely that other prediction models will do much better. Several gene expression signatures developed for prognostication showed high rates of concordance with the intrinsic subtype model in their outcome prediction, and assigned similar recurrence scores to individual tumors [14].

## Clinical Implications

Two key areas of uncertainty exist in the management of breast cancer. The first deals with the problem of identifying patients who are the most appropriate candidates for receiving adjuvant therapy. Adjuvant systemic therapy significantly improves survival of breast cancer patients. However, many patients with early-stage disease may not need adjuvant systemic therapy. Gene signature–derived tumor markers can assist in selecting those patients who will benefit most from adjuvant therapy [15]. Thus, immunohistochemistry-based subtype classification holds the promise that it can be used clinically in directing patients to the most appropriate therapy choice. The other key area is to identify those patients who are at a high risk of disease recurrence and mortality, and who will not respond to current standard therapy. Subtype classification may not be very useful in identifying those patients. Currently, we do not know whether increased metastatic potential, intrinsic drug resistance, or a combination of both is the culprit that makes a subset of primary tumors so deadly. Basal-like tumors tend to develop visceral metastases more commonly than other tumor subtypes, and it has been hypothesized that these tumors have a distinct molecular mechanism of metastatic spread that may evade detection [16]. Moreover, these tumors and triple-negative tumors may share molecular defects in their DNA repair capacity, which makes them sensitive to certain classes of DNA-damaging therapeutics. This hypothesis should be explored. Lastly, we need additional research to develop markers that can differentiate between those patients who will relapse early and those who will survive. Some studies have begun to characterize tumors from relapsing patients [12]. If successful, a molecular profile should emerge that will aid in the development of more targeted therapies that can eradicate these tumors.

## Abbreviations

ER: estrogen receptor
EGFR: epidermal growth factor receptor

## References

[1] Berry DA, Cirrincione C, Henderson IC, Citron ML, Budman DR, Goldstein LJ, Martino S, Perez EA, Muss HB, Norton L, Hudis C, Winer EP (2006). Estrogen-receptor status and outcomes of modern chemotherapy for patients with node-positive breast cancer.. JAMA.

[2] Jatoi I, Chen BE, Anderson WF, Rosenberg PS (2007). Breast cancer mortality trends in the United States according to estrogen receptor status and age at diagnosis.. J Clin Oncol.

[3] Newman LA, Griffith KA, Jatoi I, Simon MS, Crowe JP, Colditz GA (2006). Meta-analysis of survival in African American and white American patients with breast cancer: ethnicity compared with socioeconomic status.. J Clin Oncol.

[4] Carey LA, Perou CM, Livasy CA, Dressler LG, Cowan D, Conway K, Karaca G, Troester MA, Tse CK, Edmiston S, Deming SL, Geradts J, Cheang MC, Nielsen TO, Moorman PG, Earp HS, Millikan RC (2006). Race, breast cancer subtypes, and survival in the Carolina Breast Cancer Study.. JAMA.

[5] Jemal A, Siegel R, Ward E, Hao Y, Xu J, Thun MJ (2009). Cancer statistics, 2009.. CA Cancer J Clin.

[6] Blows FM, Driver KE, Schmidt MK, Broeks A, van Leeuwen FE (2010). Subtyping of Breast Cancer by Immunohistochemistry to Investigate a Relationship between Subtype and Short- and Long-Term Survival: A Collaborative Analysis of Data for 10,159 Cases from 12 Studies.. PLoS Med.

[7] Perou CM, Sorlie T, Eisen MB, Van De RM, Jeffrey SS, Rees CA, Pollack JR, Ross DT, Johnsen H, Akslen LA, Fluge O, Pergamenschikov A, Williams C, Zhu SX, Lonning PE, Borresen-Dale AL, Brown PO, Botstein D (2000). Molecular portraits of human breast tumours.. Nature.

[8] Sorlie T, Perou CM, Tibshirani R, Aas T, Geisler S, Johnsen H, Hastie T, Eisen MB, Van De RM, Jeffrey SS, Thorsen T, Quist H, Matese JC, Brown PO, Botstein D, Eystein LP, Borresen-Dale AL (2001). Gene expression patterns of breast carcinomas distinguish tumor subclasses with clinical implications.. Proc Natl Acad Sci U S A.

[9] Nalwoga H, Arnes JB, Wabinga H, Akslen LA (2010). Expression of aldehyde dehydrogenase 1 (ALDH1) is associated with basal-like markers and features of aggressive tumours in African breast cancer.. Br J Cancer.

[10] Nielsen TO, Hsu FD, Jensen K, Cheang M, Karaca G, Hu Z, Hernandez-Boussard T, Livasy C, Cowan D, Dressler L, Akslen LA, Ragaz J, Gown AM, Gilks CB, Van De RM, Perou CM (2004). Immunohistochemical and clinical characterization of the basal-like subtype of invasive breast carcinoma.. Clin Cancer Res.

[11] Mulligan AM, Pinnaduwage D, Bull SB, O'Malley FP, Andrulis IL (2008). Prognostic effect of basal-like breast cancers is time dependent: evidence from tissue microarray studies on a lymph node-negative cohort.. Clin Cancer Res.

[12] Rouzier R, Perou CM, Symmans WF, Ibrahim N, Cristofanilli M, Anderson K, Hess KR, Stec J, Ayers M, Wagner P, Morandi P, Fan C, Rabiul I, Ross JS, Hortobagyi GN, Pusztai L (2005). Breast cancer molecular subtypes respond differently to preoperative chemotherapy.. Clin Cancer Res.

[13] Carey LA, Dees EC, Sawyer L, Gatti L, Moore DT, Collichio F, Ollila DW, Sartor CI, Graham ML, Perou CM (2007). The triple negative paradox: primary tumor chemosensitivity of breast cancer subtypes.. Clin Cancer Res.

[14] Fan C, Oh DS, Wessels L, Weigelt B, Nuyten DS, Nobel AB, Van't Veer LJ, Perou CM (2006). Concordance among gene-expression-based predictors for breast cancer.. N Engl J Med.

[15] Van de Vijver MJ, He YD, Van't Veer LJ, Dai H, Hart AA, Voskuil DW, Schreiber GJ, Peterse JL, Roberts C, Marton MJ, Parrish M, Atsma D, Witteveen A, Glas A, Delahaye L, van d V, Bartelink H, Rodenhuis S, Rutgers ET, Friend SH, Bernards R (2002). A gene-expression signature as a predictor of survival in breast cancer.. N Engl J Med.

[16] Cleator S, Heller W, Coombes RC (2007). Triple-negative breast cancer: therapeutic options.. Lancet Oncol.

